# Cooperative and Opposing Functions of ANP32E and VPS72 Govern Gene Promoter Chromatin Status

**DOI:** 10.21203/rs.3.rs-8398559/v1

**Published:** 2026-01-30

**Authors:** Patrick Murphy, Shan Hua, Noah Reger, Vladyslava Sokolova, KaiLieh Huang, MaryClaire Haseley, Mystie Parker, Karli Casler, Dongyan Tan, Eric Wagner

**Affiliations:** Cornell University; Cornell University; University of Rochester; STONY BROOK UNIV; University of Rochester Medical Center; University of Rochester Medical Center; Cornell University; University of Rochester; STONY BROOK UNIV; University of Rochester

## Abstract

The histone variant H2A.Z resides within active gene promoters, but how it influences chromatin state and nucleosome stability remains poorly understood. Here, we probe two H2A.Z binding proteins with seemingly opposing function: VPS72, an SRCAP subunit that aids in H2A.Z deposition, and ANP32E, a histone chaperone which is thought to remove H2A.Z. Using functional genomics and biochemical assays, we show that VPS72 and ANP32E co-occupy active promoters yet function antagonistically. VPS72 promotes H2A.Z incorporation, acetylation, BRG1 recruitment, and transcription, whereas ANP32E constrains these features by stabilizing nucleosomes. Loss of ANP32E increases VPS72 binding, chromatin accessibility, and transcription, while co-depletion of VPS72 reverses these effects. Reconstitution assays show that ANP32E promotes nucleosome assembly and prevents DNA unwrapping, providing a mechanistic basis for *in vivo* function. Our results support a model where promoter accessibility arises from antagonistic and cooperative actions, revealing a general paradigm in which counterbalancing factors govern gene regulatory potential.

## Introduction

Eukaryotic gene expression is tightly controlled at the chromatin level through factors such as nucleosome remodelers, post-translational modifications to histones, and histone variants (Giaimo et al., 2019; Kouzarides, 2007; Skvortsova et al., 2018). These factors make up a relatively large portion of the nuclear proteome and are known to function in a diverse and often antagonistic manner, but how these various components work in concert with one another remains incompletely understood. Addressing this complexity is a central challenge in chromatin biology with broad implications in human disease (Corces et al., 2018; Dawson, 2017; Malone and Roberts, 2024; Nair and Kumar, 2012).

Several features of chromatin are known to define active or repressed gene expression states. Active promoters and enhancers are typically enriched for acetylation of histone H3 lysine 27 (H3K27ac) (Creyghton et al., 2010; Rada-Iglesias et al., 2011) and for the histone variant H2A.Z, particularly in its acetylated form (Bruce et al., 2005; Millar et al., 2006; Ren and Gorovsky, 2001). Both H3K27ac and acetylated H2A.Z (acH2A.Z) can define regions of open chromatin and facilitate transcription factor or RNA polymerase II recruitment (Bruce et al., 2005; Creyghton et al., 2010; Halley et al., 2010; Ishibashi et al., 2009; Millar et al., 2006). In contrast, H2A.Z can also localize to repressive chromatin domains, coexisting with H2K27me3 during early embryonic development, as well as in pluripotent stem cells (Creyghton et al., 2008; Hu et al., 2013; Ku et al., 2012). Furthermore, H2A.Z can also reside within or near regions marked by H3K9me3 (histone H3 lysine 9 trimethylation), a strongly repressive mark found in constitutive heterochromatin (Boyarchuk et al., 2014; Meng et al., 2023; Ryan and Tremethick, 2018). These dual associations suggest that H2A.Z fine-tines the balance between active and repressive chromatin states, though how these context-specific functions are achieved remains unclear.

One distinguishing feature of H2A.Z is its relatively high-turnover rate, due to its installation and removal from chromatin independent of DNA replication, which does not occur for canonical histones (Giaimo et al., 2019; Thambirajah et al., 2006; Wu et al., 1982; Zlatanova and Thakar, 2008). This dynamic exchange is mediated by multiple dedicated histone chaperones and remodelers, such as the SRCAP complex (which installs H2A.Z) (Kobor et al., 2004; Krogan et al., 2003) and the INO80 complex (which is thought to remove H2A.Z) (Brahma et al., 2017; Lademann et al., 2017; Papamichos-Chronakis et al., 2011; Yen et al., 2013). Additionally, the histone chaperone VPS72 (YL1), a component of the SRCAP and TIP60 complexes, directly binds the C-terminus of H2A.Z and promotes its incorporation (Cai et al., 2005; Latrick et al., 2016; Ruhl et al., 2006; Willhoft and Wigley, 2020). Another H2A.Z chaperone, ANP32E, also binds the H2A.Z C-terminus, but in contrast, it is thought to facilitate H2A.Z removal (Mao et al., 2014; Obri et al., 2014). Loss of ANP32E leads to increased promoter H2A.Z, greater chromatin accessibility, and enhanced BRG1 recruitment (Murphy et al., 2020). Strikingly, structural studies have found that ANP32E binding to H2A.Z forms an interaction domain nearly identical to that of H2A.Z when bound to VPS72 (Latrick et al., 2016; Obri et al., 2014), raising the key question: *do these two chaperones compete or cooperate to regulate H2A.Z dynamics?*

To address this question, we integrated genetics, genomics, and biochemical approaches. Our findings reveal that VPS72 and ANP32E not only co-occupy active promoters, but also exist together on chromatin within the SRCAP complex. Despite their co-residence, we find that these two factors function antagonistically – with VPS72 promoting H2A.Z incorporation, acetylation, and BRG1 recruitment while ANP32E stabilizes nucleosomes and restraining chromatin accessibility. Altogether, our data reveal that promoter homeostasis is conferred by the balanced influence of VPS72 and ANP32E, illustrating how opposing chaperones coordinate to sculpture transcriptionally competent chromatin.

## Results

### ANP32E and VPS72 bind chromatin at the same genomic locations and VPS72 binding correlates with active chromatin states

**Figure 1:**
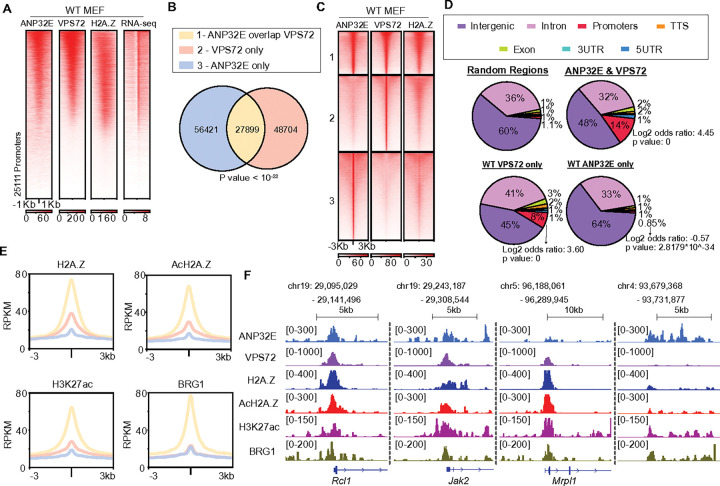
ANP32E and VPS72 bind at the same genomic regions, and VPS72 binding is positively correlated with activating chromatin features. **A**. Heatmap of RPKM normalized ANP32E, VPS72, H2A.Z enrichment signal, and gene transcription at promoters (TSS ± 1kb). Rows within heatmaps depict gene promoters, which are sorted in the same order based on enrichment. **B**. Venn diagram depicts overlap between ANP32E and VPS72 peaks. The number of peaks within each category is provided and color in the three categories are labeled as: ANP32E only, blue; ANP32E overlap with VPS72, orange; VPS72 only, pink. **C**. Heatmap of RPKM normalized ANP32E, VPS72, and H2A.Z enrichment at the peaks from three groups in 1B (center ± 3kb). Sort order is maintained across conditions, as in panel A. **D.** Pie chart depicts genomic distribution of peaks from three groups in panel 1B compared with random genomic regions (55,000 random regions with 250bp fragment length). Fisher’s exact test was used to examine the significance of overlap between promoters and peak regions in each category. The positive log2 odds ratio represents significant association, and the negative log2 odds ratio represents dissociation. **E.** Profile plots depict average RPKM enrichment for H2A.Z, AcH2A.Z, H3K27ac, and BRG1 at peak regions in the three groups. **F**. Genome browser views exemplified enrichment of ANP32E, VPS72, H2A.Z, AcH2A.Z, H3K27ac, and BRG1 (RPKM).

To begin testing whether ANP32E and VPS72 compete with each other to modulate chromatin states, we first investigated the genome-wide localization of these histone chaperones, along with the accompanying chromatin regulatory features. Using Cleavage Under Targets and Tagmentation (CUT&Tag) (Kaya-Okur et al., 2019), we mapped enrichment for H2A.Z, ANP32E, and VPS72 in primary wild type mouse embryonic fibroblasts (MEFs) (**Supplementary Fig. 1A**). Somewhat surprisingly, we found that ANP32E, VPS72, and H2A.Z were co-localized over many of the same gene promoters, with the strongest enrichment for all 3 factors occurring over highly transcribed genes (as measured by RNA-Seq) (Fig. 1A). Partitioning of promoters into quantiles (based on ANP32E enrichment) further verified co-occurrence of these proteins at actively expressed genes (**Supplementary Fig. 1B**).

We next investigated whether ANP32E and VPS72 were bound at the same genomic loci over intergenic loci. Using standard peak calling and intersection strategies, we found that many genomic loci were bound by both ANP32E and VPS72 (27,899 peaks), while numerous others were individually bound (56,421 loci for ANP32E and 48,704 for VPS72) (Fig. 1B). Interestingly, locations possessing the highest levels of VPS72 exhibited strong enrichment for H2A.Z irrespective of ANP32E binding levels (Fig. 1C), and these locations tended to occur in closer proximity to gene promoters, as compared with sites bound by only ANP32E (Fig. 1D, 1F & **Supplementary Fig. 1D**). Additional CUT&Tag measurements revealed activating chromatin features, such as histone acetylation (H3K27ac and acH2A.Z) and the chromatin remodeler BRG1, to be present over genomic locations bound by VPS72 (Fig. 1E & **Supplementary Fig. 1C**). Taken together, these results indicate that H2A.Z, ANP32E and VPS72 can bind chromatin within the same gene promoters, and high VPS72 binding is correlated with high enrichment of H2A.Z, AcH2A.Z, H3K27ac, and BRG1.

### ANP32E and VPS72 can bind chromatin at the same time

**Figure 2:**
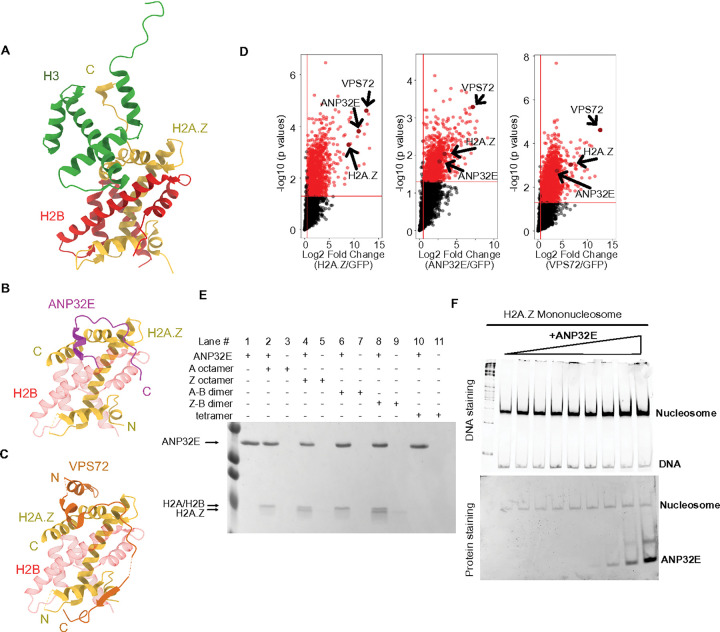
ANP32E and VPS72 bind chromatin together **A-C.** Re analysis of publicly available crystal structures for H2A.Z-containing nucleosome, ANP32E bound with H2A.Z, and VPS72 bound with H2A.Z. The same amino acid sequences from these three structures were aligned, and the structures were oriented in the same direction. The color of each protein was labeled as follows: H3 - green; H2B - red; H2A.Z - yellow; ANP32E - magenta; VPS72 - orange. **D.** Volcano plot of differentially interacting proteins from the H2A.Z ChIP-MS, ANP32E ChIP-MS, and VPS72 ChIP-MS assay compared with GFP control ChIP-MS. The proteins significantly enriched in each ChIP-MS are highlighted with red points (Log2FC > 0.5 and p values < 0.05). H2A.Z, VPS72, and ANP32E were highlighted with text arrows. **E.** *In vitro* pull-down assay using His-tagged ANP32E exhibit interaction with H2A containing octamers, H2A.Z octamers, H2A-H2B dimers, H2A.Z- H2B dimers, and histone tetramers. Migration for ANP32E, H2/H2B dimer, and H2A.Z/H2B dimers is noted. **F.** H2A.Z eviction assay were performed using H2A.Z containing mononucleosome with excess ANP32E. DNA staining indicates that nucleosomes are kept intact, and protein staining indicates that the amount of ANP32E is increased.

Our observed co-occupancy of ANP32E and VPS72 binding (via CUT&Tag) was somewhat unexpected, based on prior studies which suggested that these two factors are unable to bind H2A.Z simultaneously (Latrick et al., 2016; Obri et al., 2014). Indeed, our reanalysis of available protein structure data (Latrick et al., 2016; Obri et al., 2014; Suto et al., 2000) indicated that ANP32E and VPS72 bind to H2A.Z over precisely the same amino-acid sequence, within the C-terminal extended alpha helix portion of H2A.Z (Fig. 2A – 2C), indicating that simultaneous binding of these factors to H2A.Z itself (at least in this conformation) would be impossible. To reconcile these structural constraints with our CUT&Tag results, we considered the possibility that ANP32E and VPS72 could bind the same genomic locations at different times, or perhaps in different individual cells within a population. A third possibility is that both factors are able to bind chromatin through associated proteins or as part of multi-subunit complexes, rather than direct binding to H2A.Z.

To investigate these possibilities, we identified common and distinct interacting proteins via native Chromatin-Immunoprecipitation combined with Mass Spectrometry (ChIP-MS) (targeting endogenous ANP32E or VPS72) in HEK293T cells (**Supplementary Fig. 2A& 2B**). Significantly enriched interactors were identified by comparing results with negative control experiments using antibodies against GFP. We found that ANP32E and VPS72 co-precipitated with one another (Fig. 2D), and because our ChIP-MS was performed on mono-nucleosome size chromatin fragments (**Supplementary Fig. 2A**), this interaction likely occurs over the same individual nucleosomes simultaneously.

### ANP32E and VPS72 both bind the SRCAP complex and are predicted to interact with distinct histone surfaces

These analyses led us to reassess the manner in which ANP32E and VPS72 physically interact, and consider an alternative strategy for chromatin binding. We envisioned two separate modes by which ANP32E and VPS72 might bind chromatin. The first involves binding of ANP32E and VPS72 to the same nucleosomes, perhaps in an H2A.Z independent manner, and the second involves inclusion of both proteins within the same multi-subunit complex. Prior studies of ANP32E structure when bound to H2A.Z investigated only the C-terminal end of ANP32E (Mao et al., 2014; Obri et al., 2014), and did not consider chromatin interactions over the N-terminal portion of the protein. Interestingly, ANP32B, an ANP32E paralog, has been found to interact with histone H3 (Reilly et al., 2014; Tochio et al., 2010) and our comparison of amino-acid sequences, indicate there is a high degree of conservation over the N-terminal domain that is critical for binding to H3 (**Supplementary Fig. 2D**). Additionally, several beta-sheets that are required for histone H3 binding are present within ANP32E, and our alpha-fold3 analysis (Abramson et al., 2024) identified highly similar protein structures for ANP32E and ANP32B, with predicted binding to H3:H4 tetramers (**Supplementary Fig. 2C**). These results suggest that ANP32E may bind nucleosomes over multiple regions, including putative interactions with H3 and H2A.Z.

To further investigate the binding of ANP32E to histone octamers, tetramers, and fully formed nucleosomes, we next performed a series of *in vitro* recombinant chromatin assembly and disassembly assays utilizing zebrafish ANP32E protein, which was purified in our prior studies and is nearly identical to the human and mouse ANP32E versions (**Supplementary Fig. 2D**) (Murphy et al., 2018). As predicted, immune-precipitation assays revealed that ANP32E could interact with recombinant histone octamers and remove either canonical H2A:H2B or variant H2A.Z:H2B histone dimers upon binding (Fig. 2E). Interestingly, EMSA (electrophoretic mobility shift assays) using canonical histone substrates indicated that ANP32E was unable to bind H3:H4 tetramers (**Supplementary Fig. 2E**). Additionally, no binding was observed for re-constituted H2A.Z-containing nucleosomes, and these nucleosomes remained intact regardless of ANP32E abundance (Fig. 2F). These data indicate that ANP32E can bind histone octamers and remove either H2A or H2A.Z *in vitro*, but it cannot evict H2A.Z from chromatin in the context of recombinant nucleosomes. Considered together, these outcomes suggest that DNA unwrapping via chromatin remodeling might be necessary for removal of histone dimers.

**Figure 3:**
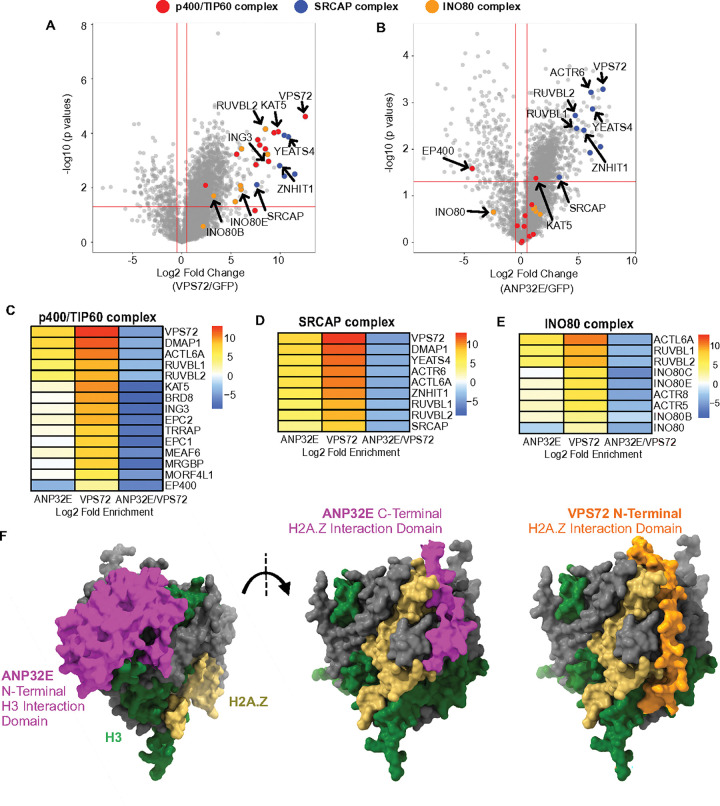
VPS72 preferentially interacts with major H2A.Z-associated chromatin factors, and ANP32E only significantly interacts with subunits of the SRCAP complex **A & B.** Volcano plots of differentially interacting proteins from the VPS72 ChIP-MS or ANP32E ChIP-MS assays compared with GFP control ChIP-MS assay. The proteins significantly enriched in VPS72 ChIP-MS are shown (Log2FC > 0.5 and p values < 0.05). The major subunits of the p400/TIP60 complex, SRCAP complex, and INO80 complex are labeled in red, blue, and orange, respectively. **C-E**. Heatmaps depict log2 fold change for major subunits of p400-TIP60, SRCAP, and INO80 complexes in ANP32E ChIP-MS, VPS72 ChIP-MS, and ANP32E ChIP-MS against VPS72 ChIP-MS. Enrichment color scale is provided, based on Log2, with red colors representing proteins that are enriched, and the blue colors represent proteins that are depleted. **F.** AlphaFold3 predicted structure of ANP32E and VPS72 bound with histone octamers. The N-terminus of ANP32E binds with the H3 surface (left), the C-terminal region of ANP32E binds with the H2A.Z surface. Right: the N-terminal of VPS72 binds with the H2A.Z surface. The color of each protein was labeled as follows: H3 - green; H2A.Z – yellow; ANP32E - magenta; VPS72 - orange.

We next investigated proteins that interacted with ANP32E and VPS72 (within our ChIP-MS data) and specifically focused on H2A.Z-associated chromatin remodeling complexes, including SRCAP, INO80, and p400/TIP60. As expected, primary subunits from both the SRCAP complex and p400/TIP60 complex were identified as enriched in VPS72 ChIP-MS assays, including SRCAP, ZNHIT11, RUVBl1, RUVBl2, VPS72, and KAT5 (Fig. 3A, 3C, 3D & **Supplementary Fig. 3A**). We also found that VPS72 interacted with some unique subunits from the INO80 remodeling complexes (Fig. 3A & 3E). ANP32E interactors included subunits from the SRCAP complex, but most proteins within the TIP60 and INO80 complexes were not enriched (Fig. 3B–3E & **Supplementary Fig. 3A**) – aside from those that are common among the SRCAP, TIP60, and INO80 complexes (Fig. 3C – 3D). Interestingly, ANP32E did not strongly interact with catalytic subunits within the TIP60 complex, EP400, and INO80 enzyme was found to be negatively enriched (Fig. 3C & 3E), indicating that ANP32E is primarily an SRCAP complex member, and the presence of ANP32E on chromatin may inhibit the binding/activity of additional H2A.Z-associated chromatin regulators.

Having found that ANP32E and VPS72 both interact with SRCAP complex sub-units and with one another, we next investigated whether alternative binding of ANP32E to nucleosomes could occur in the context of the SRCAP complex. In support of this possibility, Alpha-Fold analysis predicted that ANP32E could bind partially unwrapped nucleosomes via its N-terminal H3:H4-interacting domain (**Supplementary Fig. 3B)**, whereas the C-terminal portion of ANP32E was predicted to interact with the oppose side of the histone octamer surface where H2A.Z resides (Fig. 3F). Interestingly, this binding site almost completely overlapped with the location where VPS72 is predicted to bind the histone octamer, providing further evidence that the C-terminus of ANP32E and the N-terminus of VPS72 compete for H2A.Z binding. By overlaying these structural predictions with the known active structure of the SRCAP complex (while engaged in nucleosome remodeling) (Yu et al., 2024), we found that DNA unwrapping occurs at the precise nucleosome location where ANP32E putatively interacts with histone H3 (**Supplementary Fig. 3C**), potentially allowing for ANP32E and VPS72 to co-exist within the active SRCAP complex. These structural predictions are well aligned with our ChIP-MS data, further indicating that ANP32E and VPS72 are able bind chromatin simultaneously. Together, these results support a mechanism in which ANP32E binds chromatin via interactions with H2A.Z and with histone H3, similar to its paralog ANP32B.

### ANP32E facilitates nucleosome assembly, but binding to histone octamers leads to H2A.Z removal

**Figure 4:**
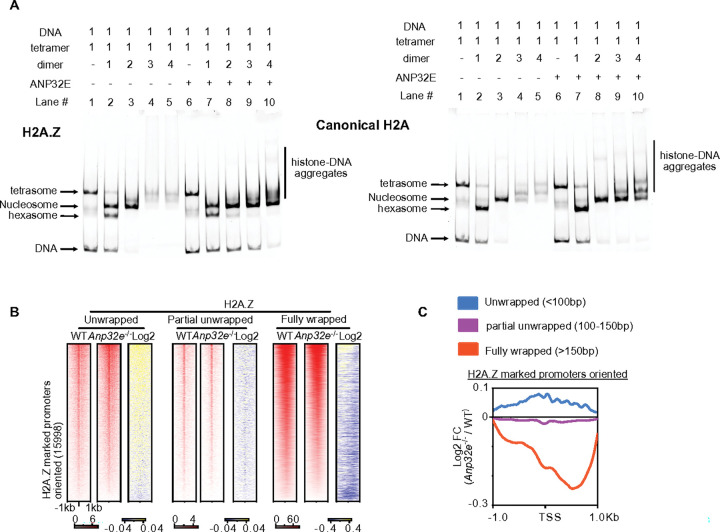
ANP32E binds with H2A or H2A.Z contained histone octamer and facilitates *in vitro* nucleosome assembly. **A.** Nucleosome assembly assay of H2A.Z and H2A with or without ANP32E. The first three lanes represent the reference ladder to indicate the migration location for histone tetramers, nucleosomes, and hexasomes. Molar ratios of each component within the reaction are indicated above each gel image. **B.** Heatmap of nucleosome wrapping at H2A.Z-marked promoters in WT and ANP32E null MEFs (TSS ± 1kb). Nucleosome wrapping is defined based on the length of DNA fragments wrapped around the H2A.Z containing nucleosomes. Unwrapped = less than 100bp; partial unwrapped = 100 to 150bp; fully wrapped = greater than 150bp. Log2 is calculated for ANP32E null MEFs over WT MEFs. **C.** Profile plot of the log2 fold change in wrapping across the three nucleosome states in ANP32E null MEFs over WT MEFs at H2A.Z-marked promoters. Unwrapped, blue; partial unwrapped, purple; fully wrapped, red.

To better understand this impact of ANP32E on nucleosome formation and DNA wrapping, we next performed chromatin assembly/disassembly assays utilizing *in vitro* recombinant histones in the presence of ANP32E protein. Although prior studies suggest that ANP32E can evict H2A.Z from chromatin (Obri et al., 2014), we found that ANP32E augmented the assembly of H2A.Z-containing nucleosomes and prevented histone aggregate formation, rather than causing disassembly or inhibiting nucleosome formation (Fig. 4A & **Supplementary Fig. 4A**). Notably, analogous outcomes were observed when assembling canonical H2A-containing nucleosomes (Fig. 4A). In prior studies, we applied a functional genomics strategy, to investigate ANP32E influences over chromatin accessibility in cultured MEFs (Murphy et al., 2020). We therefore returned to these ANP32E deficient MEFs, and applied CUT&Tag to investigate the impacts of ANP32E loss on nucleosomal DNA wrapping. Indeed, loss of ANP32E resulted in a decrease in fully wrapped nucleosomal DNA (> 150bp) within H2A.Z-enriched gene promoters, as well as an increase in unwrapped non-nucleosomal DNA (< 100bp) (Fig. 4B – 4C & **Supplementary Fig. 4B**). Reduced nucleosome wrapping was most significant over DNA sequences downstream of transcription start sites (+ 1kb), as compared with upstream DNA sequences (−1Kb) (Fig. 4C & **Supplementary Fig. 4C**). Taken together, these results provide a tactile mode in which ANP32E and VPS72 are able to bind chromatin simultaneously, and support a mechanism in which ANP32E functions to stabilize partially unwrapped nucleosomes during remodeling.

### Loss of ANP32E leads to hyper-active chromatin within gene promoters

**Figure 5:**
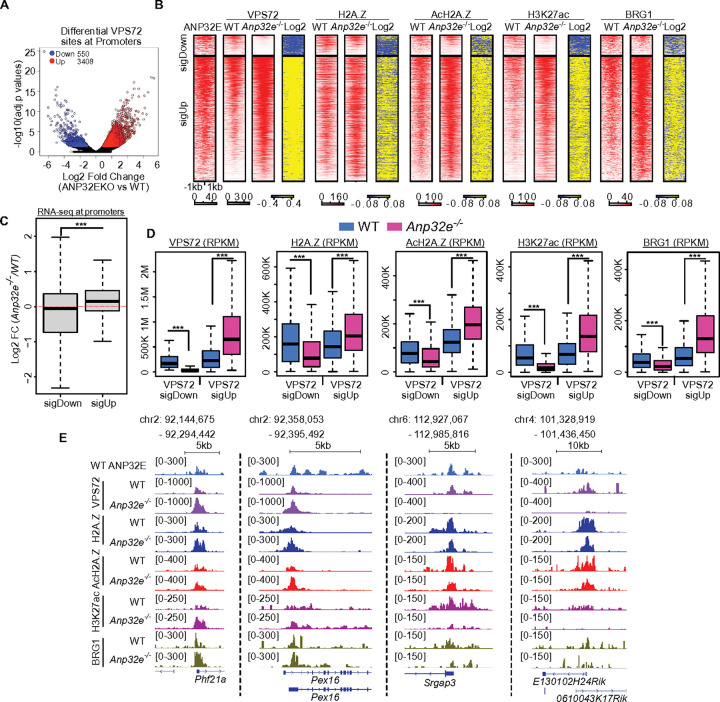
Loss of ANP32E corresponds with increased VPS72, H2A.Z, histone acetylation, and BRG1 binding at gene promoters. **A.** Volcano plot depicts differential VPS72 binding in ANP32E null MEFs versus WT MEFs at promoters. Increased VPS72 sites (3408 sites) are shown in red (log2FC > 0.5 and p. Adjusted values < 0.05). Decreased VPS72 sites (550 sites) are shown in blue (log2FC < 0.5 and p. Adjusted values < 0.05). **B**. Heatmap of ANP32E, VPS72, H2A.Z, AcH2A.Z, H3K27ac, and BRG1 enrichment in WT MEFs and ANP32E null MEFs at promoters in which VPS72 increased (SigUp) and decreased (SigDown). Log2 is calculated for the RPKM values in ANP32E null MEFs over WT MEFs. Genomic regions within all heatmaps are sorted in the same order. **C.** Boxplot of gene expression (RPKM) at increased and decreased VPS72-bound promoters. One-sided Wilcoxon rank-sum test was used to calculate p-values. ***p < 0.0001. **D.** Boxplot of RPKM normalized VPS72, H2A.Z, AcH2A.Z, H3K27ac, and BRG1 enrichment at promoters with increased and decreased VPS72 in ANP32E null MEFs. WT MEFs, blue; ANP32E null MEFs, pink. One-sided Wilcoxon rank-sum test was used to calculate p-values. ***p < 0.0001. **E**. Genome browser exemplifies ANP32E, VPS72, H2A.Z, AcH2A.Z, H3K27ac, and BRG1 enrichment (RPKM) in WT MEFs and ANP32E null MEFs.

Data from our ChIP-MS studies indicate that several H2A.Z-associated chromatin regulatory factors, such as TIP60 and INO80, are able to bind VPS72 but not ANP32E. Our CUT&Tag studies revealed that VPS72 binding was associated with high levels H2A.Z, with increased histone acetylation (H3K27ac and AcH2A.Z), and with transcriptional activation, whereas loci bound by ANP32E alone possessed ‘inactive’ chromatin (lower levels of H2A.Z and acetylation) **(**Fig. 1B–1C**)**. These observations suggest that ANP32E and VPS72 might be functionally antagonistic, and contribute to distinct chromatin states. We next speculated that maintaining ‘active’ chromatin states (possessing high H2A.Z and histone acetylation) may be dependent on this antagonism. To investigate this prospect, we performed a series of CUT&Tag experiments in MEFs lacking ANP32E (**Supplementary Fig. 5A**). Remarkably, loss of ANP32E led to an overall increase in the enrichment for VPS72 within gene promoters, 3408 loci exhibited a significantly increased enrichment while only 550 loci lost VPS72 (Fig. 5A). Additionally, promoters in which VPS72 increased also exhibited higher levels of H2A.Z, AcH2A.Z, H3K27ac, and BRG1 binding (Fig. 5B, 5D & 5E), and these promoters also tended to be more transcriptionally active (Fig. 5C). Furthermore, opposite changes in chromatin patterns occurred over gene promoters which lost VPS72 (potentially from secondary impacts). Loci in which VPS72 decrease exhibited reduced enrichment for H2A.Z, AcH2A.Z, H3K27ac, and BRG1 (Fig. 5B, 5D & 5E). Assuredly, analogous trends were observed over non-promoter regions (**Supplementary Fig. 5B – 5E**). In total, these results provide strong additional support for a mechanism in which ANP32E and VPS72 functionally antagonize one another during SRCAP remodeling to influence chromatin status of gene promoters.

### Combined depletion of VPS72 and ANP32E mitigates genomic impacts of ANP32E loss

**Figure 6:**
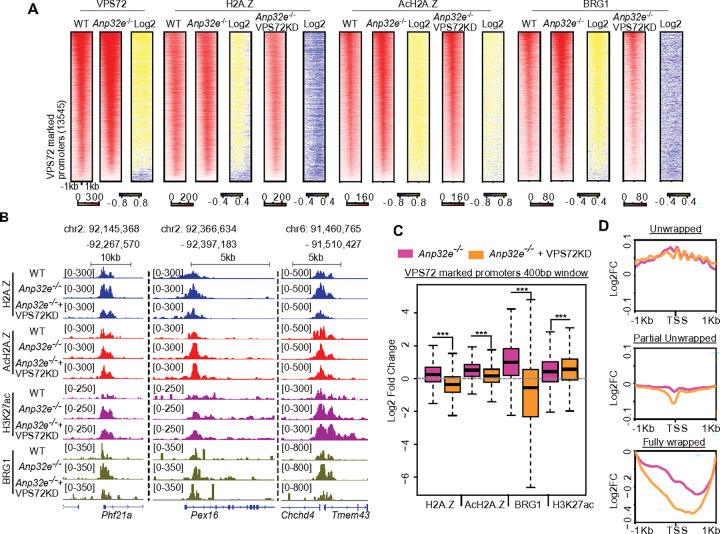
Combined loss of VPS72 and ANP32E reverses the change of epigenetic features in ANP32E null MEFs, but generates unstable and hyperactive chromatin states **A**. Heatmap of VPS72, H2A.Z, AcH2A.Z, and BRG1 binding enrichment at VPS72-marked promoters. Log2 fold change is calculated for the RPKM values in ANP32E null MEFs over WT MEFs. **B**. Genome browser exemplifies H2A.Z, AcH2A.Z, H3K27ac, and BRG1 enrichment (RPKM) in WT MEFs, ANP32E null MEFs, and ANP32E null MEFs in which VPS72 is knocked down. Combined loss of VPS72 and ANP32E reverses H2A.Z, AcH2A.Z, and BRG1 enrichment. **C**. Boxplot of log2 fold change for H2A.Z, AcH2A.Z, BRG1,and H3K27ac at VPS72-marked promoters (TSS ± 200bp). ANP32E null MEF vs WT MEFs, pink; VPS72 knockdown in ANP32E null MEFs vs WT MEFs, orange. Student’s t test was used to calculate p-values. ***p < 0.0001. **D**. Profile plot of the log2 fold change in nucleosome wrapping, as in Figure panel 4A. ANP32E null MEF vs WT MEFs, pink; VPS72 knockdown in ANP32E null MEFs vs WT MEFs, orange.

**Figure 7:**
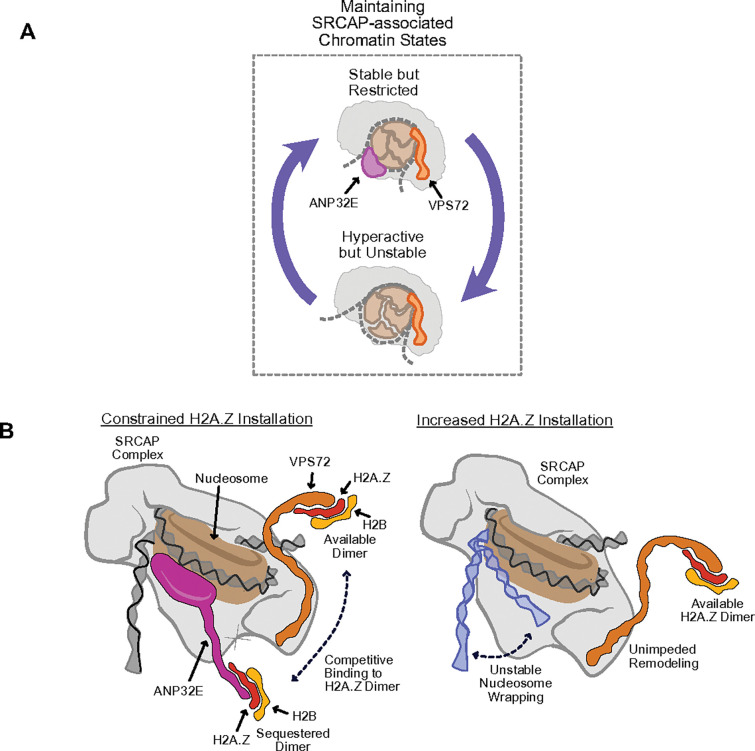
ANP32E and VPS72 are functional antagonistic, but cooperatively on chromatin state regulation **A.** A simplified model for functional interaction of ANP32E and VPS72 in the context of nucleosome stability. The presence of ANP32E and VPS72 maintain stable chromatin states, and loss of ANP32E leads to a hyperactive but unstable states, ultimately impacting nucleosome wrapping. **B**. A proposed model in which ANP32E competes with VPS72 to regulate H2A.Z installation and chromatin accessibility dynamics. The N-terminus of ANP32E binds to the octamer and stabilizes the partial unwrapped nucleosome while the SRCAP complex is actively engaged in nucleosome remodeling. Meanwhile, the C-terminus of ANP32E functions to sequester H2A.Z away from VPS72, restricting H2A.Z installation and generating a more stable chromatin state. In the absence of ANP32E, VPS72 drives increased H2A.Z installation, leading to the recruitment of other chromatin regulatory complexes, such as TIP60, p300, or BRG1, and a hyperactive but unstable chromatin state, in which DNA wrapping is more dynamic.

In the context of promoter chromatin state, our results indicate that VPS72 functions as a stimulatory chromatin factor and ANP32E as inhibitory. Having found that loss of ANP32E led to broad increases H2A.Z-associated ‘active’ chromatin states, we next investigated the degree to which these changes were a result of increased VPS72 activity. We therefore repeated our CUT&Tag measurements in MEFs which were depleted for both ANP32E and VPS72 (via genetic knockout and siRNA knockdown, respectively) (**Supplementary Fig. 6A & 6B**). As anticipated, depletion of VPS72 led to reduced enrichment for H2A.Z, AcH2A.Z, and BRG1 in MEFs already lacking ANP32E (Fig. 6A – 6C). Notably however, when we assessed the impact of VPS72 loss on nucleosome wrapping, we detected an exacerbation of unwrapping, rather than a reversal of outcomes (Fig. 6D & **Supplementary Fig. 6C – 6D**). Taken together, these results indicate that VPS72 and ANP32E functionally antagonize one another in the context of chromatin state regulation, specifically pertaining to H2A.Z incorporation, histone acetylation, and BRG1 binding, but these two factors function cooperatively to maintain stable nucleosome wrapping during SRCAP-mediated chromatin remodeling (Fig. 7).

## Discussion

Gene expression initiates when RNA polymerase engages with DNA over gene promoter regions. Throughout the genome however, nucleosome wrapping and/or chromatin modifications restricts promoter accessibility, thus limiting gene expression potential. This type of chromatin regulation plays a critical role in establishing cell identity, both during normal development and in disease contexts (Bernstein et al., 2006; Chen and Dent, 2014; Corces et al., 2018; Dawson, 2017; Hickey et al., 2022). One key factor that helps to shape promoter chromatin is the histone variant H2A.Z, which has been shown to influence gene expression and modulate nucleosome dynamics (Fan et al., 2004; Murphy et al., 2020; Mylonas et al., 2021; Weber et al., 2014). In prior studies, we and others demonstrated that H2A.Z is a critical player in the regulation of promoter chromatin states, across several eukaryotic systems (Halblander et al., 2024; Murphy et al., 2020, 2018; Raisner et al., 2005; Subramanian et al., 2013), and in the current study, we build upon these prior outcomes, to investigate mechanisms by which H2A.Z influences distinct chromatin states.

We have found that two proteins which are deeply involved in controlling H2A.Z dynamics, the chaperones ANP32E and VPS72, are structurally similar, are part of the same chromatin remodeling complex – SRCAP, and are able to bind chromatin at the same time, yet they exert opposing effects on chromatin state. Cells lacking ANP32E, exhibited increased VPS72 binding, elevated H2A.Z, increased histone acetylation, increased BRG1 recruitment, and more extensive nucleosome unwrapping, as compared with WT cells. Additionally, combined loss of ANP32E and VPS72, resulted in a reversal of these outcomes, indicative of functional antagonism. Although prior studies hint at a mechanism in which ANP32E and VPS72 cannot bind to H2A.Z at the same time (Latrick et al., 2016; Obri et al., 2014), our study reveals that both proteins can occupy the same genomic regions simultaneously, indicating that their functional opposition, and influence over H2A.Z dynamics, is not based on mutual exclusivity of binding. Rather than direct mutual exclusion, we favor a model in which ANP32E and VPS72 are present within the same multi-subunit protein complex (SRCAP) at the same time, and their presence functionally balances one another, to maintain proper H2A.Z turnover dynamics (Fig. 7).

Our structural, biochemical, and proteomics studies provided initial support for this mechanism. While ANP32E alone was able drive dissociation of H2A.Z:H2B dimer from histone octamers, it could not bind fully wrapped nucleosomes *in vitro*. We also found that the presence of ANP32E, prevented histone aggregates from forming on naked DNA, and it aided in H2A.Z containing nucleosome assembly, rather than driving histone dissociation from DNA. Together these data indicate that ANP32E is not independently responsible for H2A.Z eviction from chromatin, and instead ANP32E may partake in nucleosome stabilization. Moreover, chromatin-bound ANP32E and/or VPS72 was found to be co-bound by several SRCAP complex members, as well as other chromatin remodelers, which may act to stabilize nucleosome structure/dynamics. Additionally, our AlphaFold-based structural analyses reveal that N-terminal region of ANP32E can putatively interact with H3-H4 tetramers, and partially unwrapped nucleosomes, providing an adequate non-H2A.Z-based nucleosome interaction surface, which may allow ANP32E to stabilize nucleosomes during situations of unwrapping.

These data are better supportive of a new model for VPS72 and ANP32E function, in which both factors are present together within the SRCAP complex, but they serve opposing functions. VPS72 supports H2A.Z incorporation and chromatin remodeling, while ANP32E counters this activity to maintain a more stable, less accessible chromatin state. Further support for our model comes from our functional genomics studies. We found that promoter regions enriched for VPS72 displayed high levels of H2A.Z and histone acetylation, indicative of dynamic, transcriptionally active chromatin, while regions uniquely bound by ANP32E exhibited lower histone acetylation, and more extensive nucleosome wrapping, indicative of more stable and less active chromatin. Regions co-occupied by both ANP32E and VPS72 display intermediate features, suggestive of a balanced chromatin state in which high levels of transcription and/or nucleosome turnover can occur without catastrophic chromatin disruption. Together, these observations are well fit for our model, in which ANP32E and VPS72 function together to generate a spectrum of chromatin states via their opposing but interrelated actions.

Other factors are also known to contribute to H2A.Z regulation, such as PWWP2A, which is known to bind H2A.Z at highly transcribed gene promoters (Pünzeler et al., 2017). Much like ANP32E, loss of PWWP2A leads to increased H3K27ac and H2A.Z enrichment (Link et al., 2018). A compelling possibility is that PWWPP2A, or other histone chaperones, partake in additional antagonistic relationships to influence chromatin status. Furthermore, the histone variant H3.3 often coexists with H2A.Z within gene promoters, and this combination is known to contribute to nucleosome instability and gene activation (Jin et al., 2009; Jin and Felsenfeld, 2007). Although it is unclear whether ANP32E or VPS72 directly regulate H3.3, our findings raise the possibility that they may influence H3.3/H2A.Z nucleosome dynamics. Since H3.3 chaperones like HIRA and DAXX-ATRX regulate H3.3 patterns over distinct genomic loci (Shi et al., 2017), it remains possible they too antagonize one another, much like ANP32E and VPS72, to influence chromatin dynamics/status from a completely separate pathway.

These findings lead us to a broader conceptual framework: chromatin regulation may often rely on mutual antagonism between opposing factors that maintain balance across genomic loci. ANP32E and VPS72 serve as a prominent example of this principle. While they oppose one another functionally, they are both essential for proper chromatin regulation. Loss of either leads to imbalance and dysregulation. This concept is akin to “Yin and Yang” – opposing forces that work together to create harmony. Similarly, they resemble a married couple, each bringing different strengths and perspectives to a shared goal. In sum, our study provides compelling evidence that ANP32E and VPS72 not only regulate H2A.Z dynamics through antagonistic actions but also cooperatively shape context-specific chromatin states. Their interaction reflects a broader biological principle – opposing forces often work together to maintain homeostasis, flexibility, and function.

## Resource Availability

### Lead Contact

Further information and requests for reagents or resources should be directed to (and will be fulfilled by) the Lead Contact Patrick J. Murphy (pjm249@cornell.edu).

## Methods

### Data and Resource Availability

Further information and requests for reagents or resources should be directed to (and will be fulfilled by) the Lead Contact Patrick J. Murphy (pjm249@cornell.edu). Sequencing data generated in this study can be found at the European Nucleotide Archive (ENA) under study accession number PRJEB100622. Mass-Spec data generated in this study can be accessed via PRIDE, accession number PXD069562.

### Experimental Model and Study Participant Details

#### Murine Cell Culture

Primary wild type C57B6 and ANP32E null C57B6 mouse embryonic fibroblasts (MEFs) were acquired from embryonic day 10.5 mouse embryos, as noted in our prior study (Murphy et al., 2020). DMEM (Thermo Fisher #11995065) supplemented with 10% FBS (Thermo Fisher #26140079) and 1% penicillin-streptomycin (Thermo Fisher #15140122) was used to culture MEFs. Cells were grown at 37°C with 5% CO_2_. MEFs at passages 10–14 were used for all assays.

## Methods Details

### VPS72 siRNA knockdown

Lipofectamine RNAiMAX (Thermo Fisher #13778075), a non-targeting siRNA pool (Dharmacon #D-001810–10-05), and a pool of 3 VPS72 siRNA (sc-155224) were used to transfect ANP32E null MEFs. The non-targeting pool and VPS72 siRNA pool were diluted to 145 pmol in Opti-MEM (Thermo Scientific #31985062). RNAiMAX was diluted in Opti-MEM. A combination of either a non-targeting pool or VPS72 siRNA with RNAiMAX was incubated at room temperature for 8 mins and was used for transfection. The transfected cells were incubated for 48 hours at 37 °C.

### Western blotting

Approximately 500,000 cells were used for western blots. Cultured cells were washed with 1mL cold 1X PBS, spun down at 600×g for 3 minutes, and decanted the PBS. The cell pellets were lysed with 500 μL cold 1X RIPA lysis buffer (10X stock Millipore Sigma #20–188) and incubated on ice for 5 minutes. The cells were then sonicated using 25% amplitude with 30s on and 30s off for a total of four times to dissociate chromatin bound proteins within nuclei. During sonication, cells were chilled on ice after two pulses to prevent overheating. The cell lysates were centrifuged at 13,000g at 4 °C for 5 mins, and the supernatant was collected. Protein concentration was measured using Bio-Rad protein assay dye reagent (Bio-Rad # 5000006). Serial dilution of BSA solution (2 mg/ml, 1.5 mg/mL, 1 mg/mL, 0.75 mg/mL, 0.5 mg/mL, 0.25 mg/mL, 0.125 mg/mL) was made for the standard curve. A combination of 5 μL protein lysates or diluted BSA with 800 μL diluted Bio-Rad protein assay dye reagent was incubated at room temperature for 10 minutes. The measured proteins lysates were mixed with 2X Laemmli buffer (Bio-Rad #1610737EDU) and boiled at 95°C for 5 minutes before loading to protein gels. The 12% separating gel (40% acrylamide mix, 1.5 M Tris pH 8.8, 10% SDS, 10% APS, TEMED and water) and 5% stacking gel (40% acrylamide mix, 0.5 M Tris pH 6.8, 10% SDS, 10% APS, TEMED and water) were used for gel running. Approximately 40 μL of protein lysates was loaded into each well and run for one hour at 150 V using 1x SDS running buffer. The 1x transfer buffer (3.03 g Tris Base, 14.4 g Glycine, 200 ml methanol, and water up to 1L) was used to transfer proteins from the gel to the nitrocellulose membrane at 350 mA for one hour. The blocking buffer (0.5g dried milk in 10 ml 1X TBS) was used to prevent non-specific binding. VPS72 (Thermo Fisher #15143–1-AP) and H1 (Active Motif #39707) primary antibodies were diluted in 5% non-fat dried milk in TBST, and incubated at 4 °C overnight. The VPS72 antibody was applied at 1:500 dilution and H1 was 1:1000 dilution. The following day, the membrane was washed three times using TBST for 15 minutes each. The Starbright anti-mouse (Bio-Rad #12004158) and anti-rabbit secondary antibodies (Bio-Rad #12005869) in 5% non-fat dried milk in TBS were added to the membrane and incubated at room temperature for one hour. TBS was used for washes of the membrane before imaging.

### Co-Immunoprecipitation with samples preparation

A confluent 10 cm plate of HEK293T cells was collected for each replicate of ChIP-MS. The cells were washed in 1 mL cold 1X PBS, spun down at 600×g for 3 mins, then the liquid was removed and wash was repeated. The cell pellet was resuspended in 1.5 mL cold IP lysis buffer (50 mM Tris pH8, 100 mM NaCl, 10% Glycerol, 1% NP-40, 0.2 mM EDTA, 0.5 mM DTT, 0.5 mM PMSF). Cell lysates were mixed with 2 μL Benzonase (Sigma #E1014) and 1.5 μL 1M MgCl2 and were rotated at 4°C for 4 hours. The supernatant was collected after spinning down at max speeds at 4°C for 3 minutes. Approximately 20 μL of supernatant was saved as input for western blotting. The remaining portion of the solution was incubated with primary antibodies at 4°C overnight. The following day, protein A/G beads (Thermo Fisher #88802) were washed with 1 mL cold IP wash buffer (20 mM HEPES pH7.4, 100 mM KCl, 10% Glycerol, 0.1% NP-40) for a total of three cycles. Beads were resuspended in 1 mL cold IP wash buffer and aliquot of 30 μL in each microfuge tube. The tubes were placed on a magnetic stand, and the wash buffer was removed from the beads. The antibody lysates were added to the beads and rotated at 4°C for 2 hours. The beads with lysates were then washed in 1 mL ice cold IP wash buffer and rotated at 4°C for 5 minutes a total of four times. At the fifth wash, the beads were resuspended in 1 mL cold 50 mM HEPES pH 7.4 and transferred to the new tube. The supernatant was then removed, and the beads were stored in −80 °C and used for Mass spectrometry and western blotting. Antibodies against endogenous H2A.Z (Active Motif #39113), ANP32E (Invitrogen #PA5–42860), VPS72 (Thermo Fisher #15143–1-AP), and GFP (Fisher Scientific #A6455) were used for pulled down from cell lysates. Gel electrophoresis was performed to check the chromatin fragment length after Benzonase digestion following different time points, and 4 hours digestion was used in the current study. The S-strap protein digestion experiment was performed before assessing mass spectrometry. The beads from −80 °C were resuspended in 32 μL 50mM TEAB with 5% SDS (1M TEAB Thermo Fisher #90114, 10% SDS Invitrogen #AM9822) and were boiled for 5 minutes. The supernatant was collected into new tube after centrifugation, and then 2.2 μL of 25mM DTT/TEAB (1M TEAB, DTT Thermo Fisher #R0862) was added to the samples and incubated at 55°C with 800RPM spin speed for one hour. Samples were then placed at room temperature. 2.4 μL of 125mM IAA/TEAB (1M TEAB, 23.1mg Iodoacetamide Thermo Fisher #AC122270250) was added to the samples, which were keep in the dark for 30 minutes at room temperature. 3.3 μL of 12% phophoric acid (85% Phosphoric acid Fisher #A260–500) and 198 μL 90% Methanol/TEAB (100% Methanol Fisher #BP1105–4, 1M TEAB) were added to the samples, repectively, and the samples were resuspended thourouly. 120 μL of sample was transferred into S-trap column (Protifi #C02-micro) each time and spun it down at room temperture with 4000g for 1 minute. 165 μL of 90% Methanol/TEAB was used to wash the samples with total two times. Additional centrifugation was performed to remove any residual wash buffer and the S-trap column was transferrred to a new 1.5mL tube. 20 μL Trypsin/TEAB (1M TEAB, Trypsin Pierce #PI90058) was added to the column and it was kept at room temperature for 5 minutes. The column was then placed in 37°C waterbath for 20 hours. 20 μL 0.1% TFA (Thermo Fisher #LS119–500) in water was added to the samples and the samples were then centrifuged at room temperture with 4000g for 1 minute. 40 μL of 50% Acetonitrille (Fisher #LS121–500) in water and samples were collected after spinning them down at room temperture with 4000g for 2 minutes. The samples were stored in −80°C until mass spectormetry.

### Mass Spectrometry

The digested peptides were injected onto a 75 μm × 2 cm trap column (Thermo Fisher), and a Vanquish Neo UHPLC (Thermo Fisher) attached to an Orbitrap Astral mass spectrometer was used to refocus the peptides on an Aurora Elite 75 μm × 15 cm C18 column (IonOpticks). Ions were added to the mass spectrometer, which was operated at 2 kV using Easy-Spray source. The concentration of solvent B (0.1% formic acid in 80% acetonitrile) started with 1% and increased to 5% in 0.1 minutes. The concentration of solvent B continues to increase to 30% in 12.1 minutes, 40% in 0.7 minutes, and 99% in 0.1 minutes at the end. The column was washed using 99% solvent B for 2 minutes with total 15 minutes runtime. In each mass spectrometry run, 1% solvent B was used to re-equilibrated the column before the next injection. A data-independent acquisition (DIA) mode was set for the Orbitrap Astral and MSI scans were acquired with 240,000 resolution. 5 ms with a range of 380–980 m/z was used for the maximum injection time. DIA MS2 scans was used in the Atral mass analyzer and 6 ms maximum injection time was used with variable windowing (4Da – 380–750 m/z and 6Da – 750–980 m/z). The HCD collision energy and the normalized AGC were set as 28% and 500%, respectively. A range of 150–2000 m/z with 0.6 seconds cycle time was set to obtain fragment ions.

### CUT&Tag and library preparation

The CUT&Tag protocol was used from Dr. Steven Henikoff’s group (version 2 from protocols.io https://www.protocols.io/view/bench-top-cut-amp-tag-kqdg34qdpl25/v2?version_warning=no). Fresh or cryopreserved cells were used for CUT&Tag. Concanavalin A-coated beads (Fisher Scientific #BP531) were these to wash samples, using 1.5 mL wash buffer (1M HEPES pH7.5, 5M NaCl, 2M Spermidine, protease inhibitor). Washed beads were resuspended in binding buffer (1M HEPES pH7.5, 1M KCl, 1M CaCl2, 1M MnCl2). Cells were washed twice using 1 volume wash buffer at room temperature. Beads slurry was added dropwise into cells, which were then rotated for 10 mins. Beads were resuspended in cold antibody buffer (wash buffer, 5% digitonin, 30% BSA, 0.5M EDTA) and were evenly split into new tubes with 50 μL each. The primary antibody was then mixed with beads and incubated on a nutator overnight at 4°C. The next day, the tubes were placed on magnetic stands, and the liquid was removed. A secondary antibody with Dig-wash buffer (100 μL per samples) was added and was incubated on a nutator at room temperature for 1 hour. Dig-wash buffer was used to wash the beads three times in total. The beads were resuspended in 100 μL of pA-Tn5 in Dig-300 buffer and were incubated on a nutator for 1 hour. The beads were then washed using dig-300 buffer three times in total and then resuspended in 300 μL tagmentation buffer (Dig-300 buffer with 1M MgCl2). The solution was incubated at 37°C for 1 hour. Stop tagmentation buffer (10 μL 0.5M EDTA, 3 μL 10% SDS and 2.5 μL 20 mg/mL Proteinase K) was added and was incubated at 50°C for 1 hour. After tagmentation was stopped, phenol chloroform was added and samples were centrifuged at maximum speed for 3 mins. Chloroform was added in the same tube and inverted the tubes ten times and centrifuged at maximum speed for 3 mins. The aqueous layer was transferred into the tube contained 750 μL 100% ethanol and 1uL glycoblue (Thermo Scientific #am9516), and the sample was centrifuged at 4°C 16,000×g for 15 minutes. Liquid was removed and DNA pellet was rinsed with 1mL 100% ethanol. The DNA pellet was drained on paper towel and air dry for 5 minutes. The DNA was resuspended in 30uL water with RNaseA (1:400) (Thermo Fisher #12091–021) and was incubated at 37°C for 10 mins to dissolving DNA. Primary antibodies H2A.Z (Active Motif #39113), ANP32E (Invitrogen #PA5–42860), VPS72 (Thermo Fisher #15143–1-AP), AcH2A.Z (Millipore Sigma #ABE1363), BRG1 (Abcam #ab110641), and H3K27ac (Active Motif #39133) were used. The concentration of antibodies against H2A.Z, AcH2A.Z, and H3K27ac was 1:50 dilution, and the concentration of ANP32E, VPS72, and BRG1 antibodies was 1:25 dilution. The guinea pig anti-rabbit secondary antibody (Novus Biologicals #NBP172763) was used, and the concentration was 1:100 dilution. Adapter loaded pA-Tn5 was used at a concentration of 1:100 dilution. NEBNext High-Fidelity 2x PCR Master Mix (NEB #M0541L) was used to prepare CUT&Tag DNA libraries, which were cleaned up using 1.1 volume of AMpure XP beads (Fisher Scientific #NC9933872). Samples were then pooled together for sequencing. CUT&Tag DNA libraries were sequenced using NovaSeqX 150bp pair-ended with an estimated 40M reads per sample.

### Published protein structure and Alpha fold predication

Publicly available PDB files of H2A.Z contained nucleosome, human ANP32E-H2A.Z complex, and human YL1-H2A.Z complex were 1F66 (Suto et al., 2000), 4CAY (Obri et al., 2014), and 5FUG (Latrick et al., 2016), respectively.Anp32e tetramer, Anp32b tetramer, Anp32e octamer, and VPS72 octamer predicted structures were generated using AlphaFold3 with the following Uniprot sequences: Q9BTT0 (Anp32e), Q92688 (Anp32b), Q15906 (VPS72), Q93077 (H2A), P62807 (H2B), P68431 (H3.1), P62805 (H4), and P0C0S5 (H2A.Z). The following PDB files were used to generate SRCAP models: 8X15 (SRCAP-C apo state) and 8X1C (SRCAP-C ADP-bound state) (Yu et al., 2024). Images were generated using ChimeraX (v1.7.1).

### Multiple Protein Sequences Alignment

ANP32B and ANP32E protein sequence in *Homo Sapiens*, *Mus Musculus*, *Danio Rerio*, and *Xenopus Tropicalis* were downloaded as FASTA files from NCBI. The FASTA files were imported into MEGA 11 (v11.0.13), and MUSCLE alignment was used to align multiple protein sequences. The combined aligned protein sequences were saved as a FASTA file. The T-COFFEE Multiple Sequence Alignment website was used to convert the combined FASTA file into a ClustalW file. The ClustalW file was imported into Jalview (v2.11.4.1) and generated conservation protein alignment images. The color was based on the amino acids percentage identity.

## Biochemistry Analysis

### DNA

The plasmid containing twelve tandem repeats of 167-bp Widom 601 DNA sequence was a gift from professor Craig Peterson. Large scale plasmid production was performed as previously described (Dyer et al., 2003), with the following modifications: EcoRV (NEB) was used to excise the single-repeat 601 DNA from the plasmid backbone, and MonoQ (Cytiva) anion exchange chromatography was employed as the final purification step following polyethylene glycol precipitation. The sequences of the 167-bp Widom 601 sequence is as the following with the 601 DNA sequence underlined:

ATCCCGCCCTGGAGAATCCCGGTGCCGAGGCCGCTCAATTGGTCGTAGACAGCTCTAGCACCGCTTAAACGCACGTACGCGCTGTCCCCCGCGTTTTAACCGCCAAGGGGATTACTCCCTAGTCTCCAGGCACGTGTCAGATATATACATCCTGTGCATGACTAGAT

### Histone Purification

*Xenopus laevis* histones H2A, H2B, and H4 and mouse H2A.Z variant histone were expressed in *E. coli* BL21 (DE3) competent cells (Invitrogen). *Xenopus laevis* H3 was expressed in BL21 (DE3) pLysE competent cells (Invitrogen). All histones purification were conducted following the standard protocol (Dyer et al., 2003). To reconstitute H2A-H2B dimer or H2A.Z-H2B dimer, 1:1 ratio of respective histones was mixed and incubated for 2 hour at 4°C in unfolding buffer [7 M guanidine HCl, 20 mM Tris (pH 7.5), and 10 mM DTT], followed by dialysis against at least three changes of refolding buffer [10 mM Tris (pH 7.5), 1 mM EDTA, 2 M NaCl and 1 mM DTT]. The same procedure was used to reconstitute H3-H4 tetramer, by mixing 1:1 ratio of H3 and H4. Dimer or tetramer was concentrated and purified via gel filtration chromatography using a Superdex200 increase 10/300 column (GE healthcare).

### Nucleosome assembly assay

Dimer and tetramer were mixed with 167-bp DNA in high salt buffer [10 mM Tris (pH 8.0), 2 mM EDTA, 2 M NaCl, and 2 mM 2-mercaptoethanol (βME)] with or without Anp32E. The mixture was dialyzed overnight into low-salt buffer [10 mM Tris (pH 8.0), 2 mM EDTA, 5 mM NaCl, and 2 mM βME] at 4°C, gradually or directly. The reaction was loaded on an 4% Native-PAGE gel in 1x TBE and run at 100 V for 60 min at 4°C. After electrophoresis, the gel was stained with SYBR-GOLD (GoldBio) for 10 min and then imaged on a Typhoon imager (Cytiva).

### Anp32E pulldown assay

Anp32E-10xHIS was mixed with H3-H4 tetramer, H2A-H2B, H2A.Z-H2B dimer, H2A octamer, and H2A.Z octamer respectively, in high salt buffer. The mixture was gradually dialyzed into 600 ml reaction buffer [10 mM Tris (pH 8.0), 2 mM EDTA, 150 mM NaCl, and 2 mM βME] at 4°C, gradually for 16h. The reactions were centrifuged to remove precipitations. The supernatant was then mixed with 10ul Ni-NTA resin in a total reaction volume of 100ul for an hour at 4°C with continuous rotation. The resin was pelleted by centrifugation, and unbound proteins were removed by three washes with washing buffer [10 mM Tris (pH 8.0), 2 mM EDTA, 150 mM NaCl, 10mM imidazole]. Bound proteins were eluted with elution buffer [10 mM Tris (pH 8.0), 2 mM EDTA, 150 mM NaCl, 250mM imidazole]. Eluted samples were mixed with Laemmli sample buffer, boiled for 10 minutes and resolved on 15% SDS-PAGE.

### H2A.Z eviction experiment

H2A.Z nucleosomes were mixed with increasing amounts of Anp32E and incubated at room temperature. Samples were resolved on a 4% Native-PAGE gel at 4°C (100 V, 60 min, 1× TBE). The gel was stained with SYBR-GOLD (GoldBio) for 10 minutes with shaking and imaged using a Typhoon imager (Cytiva).

### EMSA Assay

10uM of chaperone were mixed with increasing amount of either H2A/H2B dimer of H3/H4 tetramer (0, 2, 5, 10 uM) in 20 ul and dialyzed into binding buffer (50mM Tris-HCl pH 7.5, 150 mM NaCl, 1mM EDTA, 1mM DTT). Samples were run on 4% Native-PAGE (100V, 1.5 h) and stained with Coomassie.

### Bioinformatic Analysis

#### Alignment and Peakcalling

FastQC (v0.11.8) was used for quality control analysis of fastq files. The adapter sequence was trimmed at the 3’end of reads in each pair using cutadapt (vb1). Bowtie2 (v2.3.5.1) was used to align all CUT&Tag fastq files to the mouse reference genome Mm10. Picard (v2.12.0) SortSam was used to convert SAM files to BAM files. Duplication reads were removed using Picard MarkDuplicates. Picard BuildBamIndex was used to build an index file for each bam file. Deeptools (v3.5.1) bamCoverage was used to convert bam files to bigwig files with read count normalization (--binSize 10, --normalizeUsing RPKM, --extendReads). Three technical replicates bigwig files were merged as a bedGraph file using UCSC bigWigMerge (v2.10). The normalized bedGraph files were converted into merged bigwig files using UCSC bedGraphToBigWig (v2.10). IGV (v2.8.9) was used to visualize bigwig files and generate genome tracks. Deeptools bigwigCompare was used to generate log2 fold change of RPKM values bigwig files. Macs2 bdgpeakcall (v2.2.6) (-c -g -l) was used to perform peakcalling on ANP32E and VPS72 CUT&Tag data. Bedtools (v2.30.0) intersect was used to find the overlap regions between ANP32E and VPS72 or their individual occupied regions. The random regions (bed file format) were generated using Bedtools random. Bedtools Fisher was used to perform Fisher’s exact test on the overlap of promoters and interaction peaks (ANP32E & VPS72) or their individual peaks.

#### Define Nucleosome Wrapping Status

H2A.Z CUT&Tag data in WT, ANP32E null, ANP32E null with VPS72 siRNA were used to classify nucleosome wrapping statues. The three technical replicates of WT, ANP32E null, and ANP32E null with VPS72 siRNA bam files (without duplication reads) were merged using samtools (v1.9) merge, respectively. Merged bam files were sorted by read name using bedtools (v2.30.0) sort (-n), and bedtools pairtobed was used to report the reads from both pairs in merged bam files overlapping with promoters and reporting as a new bam file. The samtools view (-b, -s) was used to subset each new bam file with equal mapped reads of H2A.Z over promoters, enabling us to correct for differences in sequence coverage depth. The new bam file was then sorted by coordinate using the samtools sort default setting. Deeptools (v3.5.1) alignmentSieve was used to subset different fragment sizes on bam files to classify unwrapped (less 100bp), partially unwrapped (100 -150bp), and fully wrapped nucleosomes (greater 150bp). Picard BuildBamIndex was used to build index files, and bamCoverage was used to convert bam files to new bigwig files without normalization. The read count boxplot was generated in R studio, and the one-tailed Wilcox test (x, y, alternative = “greater” or “less”) was used for statistical significance.

#### Plotting and visualization

For ChIP-MS data, volcano plots were generated using ggplot2 (v3.5.2) in R studio (v2025.05.1+513) to assess differential changes. The defined proteins of interest were selected using the R packages ggrepel (v0.9.6) and dplyr (v1.1.4). The heatmap of each chromatin remodeling complex subunit was generated using pheatmap (v1.0.13) without clustering. The scatterplot of technical replicates was generated using default base R settings (plot function) with the Spearman correlation test in R studio. For CUT&Tag data, the Venn Diagram was generated using the package eulerr (v7.0.2) in R Studio. Homer peak annotation (annotatePeaks.pl) with a default setting was used to find the genomic distribution of the peaks (bed file format), and a pie chart was generated in R Studio. Heatmaps were generated using Deeptools computeMatrix and plotHeatmap. The average signal of each mark was plotted using plotProfile. Deeptools multibigwigSummary and plotCorrelation was used to generate a Pearson correlation plot of all the technical replicates for each histone mark. The RPKM values for each chromatin mark over promoters or union peak sets were generated as a tab-delimited table from computeMatrix, which was used to generate boxplots with a default setting in base R studio. One-sided Wilcoxon rank sum tests (x, y, alternative = “greater” or “less”) was used for assessing statistical significance. Package Diffbind was used to analyze differential VPS72 binding, and volcano plots were generated in R studio with a default base setting. Affinity Designer was used to adjust font sizes, color and labels.

## Statistical Methods

One-sided Wilcoxon rank sum tests were used to calculate p values for all the boxplots. Asterisks represent the significant differences across each sample. The asterisks key represents: * < 0.01, ** < 0.001, *** < 0.0001. For boxplots, the black lines in the box represent median values. Boxes represent interquartiles between 25% and 75%, whiskers represent outer quartiles (0–25% and 75–100%). The figure legends also include statistical parameters. Pearson correlation test was used for plotting correlational heatmap. Adjusted p values applied to multiple test correction for all differential enrichment tests.

## Resource Availability

### Lead Contact

Further information and requests for reagents or resources should be directed to (and will be fulfilled by) the Lead Contact Patrick J. Murphy (pjm249@cornell.edu).

### Data and Code Availability

- Sequencing data generated in this study have been deposited in the European Nucleotide Archive (ENA) under study accession number PRJEB100622 and are publicly available as of date of publication. Accession numbers are listed in the key resources table. Mass-Spec data generated in this study can be accessed via PRIDE, accession number PXD069562 is listed in the key resources table.

### Material Availability

This study did not generate new unique reagents.

## Supplementary Material

Supplementary Files

This is a list of supplementary files associated with this preprint. Click to download.

• SupplementaryFigureswithFigureLegends.pdf

## Key Points

- Promoter chromatin state is controlled by opposing histone chaperones
- H2A.Z influence over chromatin states occurs via its chaperones
- VPS72 and ANP32E operate in both an antagonistic and cooperative manner
- Nucleosome stability and DNA wrapping are influenced by H2A.Z dynamics

## References

[1] Abramson J., Adler J., Dunger J., Evans R., Green T., Pritzel A., Ronneberger O., Willmore L., Ballard A.J., Bambrick J., Bodenstein S.W., Evans D.A., Hung C.-C., O’Neill M., Reiman D., Tunyasuvunakool K., Wu Z., Žemgulytė A., Arvaniti E., Beattie C., Bertolli O., Bridgland A., Cherepanov A., Congreve M., Cowen-Rivers A.I., Cowie A., Figurnov M., Fuchs F.B., Gladman H., Jain R., Khan Y.A., Low C.M.R., Perlin K., Potapenko A., Savy P., Singh S., Stecula A., Thillaisundaram A., Tong C., Yakneen S., Zhong E.D., Zielinski M., Žídek A., Bapst V., Kohli P., Jaderberg M., Hassabis D., Jumper J.M., 2024. Accurate structure prediction of biomolecular interactions with AlphaFold 3. Nature 630, 493–500. 10.1038/s41586-024-07487-w 38718835 PMC11168924

[2] Bernstein B.E., Mikkelsen T.S., Xie X., Kamal M., Huebert D.J., Cuff J., Fry B., Meissner A., Wernig M., Plath K., Jaenisch R., Wagschal A., Feil R., Schreiber S.L., Lander E.S., 2006. A Bivalent Chromatin Structure Marks Key Developmental Genes in Embryonic Stem Cells. Cell 125, 315–326. 10.1016/j.cell.2006.02.041 16630819

[3] Boyarchuk E., Filipescu D., Vassias I., Cantaloube S., Almouzni G., 2014. The histone variant composition of centromeres is controlled by the pericentric heterochromatin state during the cell cycle. J. Cell Sci. 127, 3347–3359. 10.1242/jcs.148189 24906798

[4] Brahma S., Udugama M.I., Kim J., Hada A., Bhardwaj S.K., Hailu S.G., Lee T.-H., Bartholomew B., 2017. INO80 exchanges H2A.Z for H2A by translocating on DNA proximal to histone dimers. Nat. Commun. 8, 15616. 10.1038/ncomms15616 28604691 PMC5472786

[5] Bruce K., Myers F.A., Mantouvalou E., Lefevre P., Greaves I., Bonifer C., Tremethick D.J., Thorne A.W., Crane-Robinson C., 2005. The replacement histone H2A.Z in a hyperacetylated form is a feature of active genes in the chicken. Nucleic Acids Res. 33, 5633–5639. 10.1093/nar/gki874 16204459 PMC1243646

[6] Cai Y., Jin J., Florens L., Swanson S.K., Kusch T., Li B., Workman J.L., Washburn M.P., Conaway R.C., Conaway J.W., 2005. The Mammalian YL1 Protein Is a Shared Subunit of the TRRAP/TIP60 Histone Acetyltransferase and SRCAP Complexes*. J. Biol. Chem. 280, 13665–13670. 10.1074/jbc.M500001200 15647280

[7] Chen T., Dent S.Y.R., 2014. Chromatin modifiers and remodellers: regulators of cellular differentiation. Nat. Rev. Genet. 15, 93–106. 10.1038/nrg3607 24366184 PMC3999985

[8] Corces M.R., Granja J.M., Shams S., Louie B.H., Seoane J.A., Zhou W., Silva T.C., Groeneveld C., Wong C.K., Cho S.W., Satpathy A.T., Mumbach M.R., Hoadley K.A., Robertson A.G., Sheffield N.C., Felau I., Castro M.A.A., Berman B.P., Staudt L.M., Zenklusen J.C., Laird P.W., Curtis C., The Cancer Genome Atlas Analysis Network, Greenleaf W.J., Chang H.Y., 2018. The chromatin accessibility landscape of primary human cancers. Science 362, eaav1898. 10.1126/science.aav1898 30361341 PMC6408149

[9] Creyghton M.P., Cheng A.W., Welstead G.G., Kooistra T., Carey B.W., Steine E.J., Hanna J., Lodato M.A., Frampton G.M., Sharp P.A., Boyer L.A., Young R.A., Jaenisch R., 2010. Histone H3K27ac separates active from poised enhancers and predicts developmental state. Proc. Natl. Acad. Sci. 107, 21931–21936. 10.1073/pnas.1016071107 21106759 PMC3003124

[10] Creyghton M.P., Markoulaki S., Levine S.S., Hanna J., Lodato M.A., Sha K., Young R.A., Jaenisch R., Boyer L.A., 2008. H2AZ Is Enriched at Polycomb Complex Target Genes in ES Cells and Is Necessary for Lineage Commitment. Cell 135, 649–661. 10.1016/j.cell.2008.09.056 18992931 PMC2853257

[11] Dawson M.A., 2017. The cancer epigenome: Concepts, challenges, and therapeutic opportunities. Science 355, 1147–1152. 10.1126/science.aam7304 28302822

[12] Dyer P.N., Edayathumangalam R.S., White C.L., Bao Y., Chakravarthy S., Muthurajan U.M., Luger K., 2003. Reconstitution of Nucleosome Core Particles from Recombinant Histones and DNA, in: Methods in Enzymology, Chromatin and Chromatin Remodeling Enzymes, Part A. Academic Press, pp. 23–44. 10.1016/S0076-6879(03)75002-2 14870657

[13] Fan J.Y., Rangasamy D., Luger K., Tremethick D.J., 2004. H2A.Z Alters the Nucleosome Surface to Promote HP1α-Mediated Chromatin Fiber Folding. Mol. Cell 16, 655–661. 10.1016/j.molcel.2004.10.023 15546624

[14] Giaimo B.D., Ferrante F., Herchenröther A., Hake S.B., Borggrefe T., 2019. The histone variant H2A.Z in gene regulation. Epigenetics Chromatin 12, 37. 10.1186/s13072-019-0274-9 31200754 PMC6570943

[15] Halblander F.N., Meng F.W., Murphy P.J., 2024. Anp32e protects against accumulation of H2A.Z at Sox motif containing promoters during zebrafish gastrulation. Dev. Biol. 507, 34–43. 10.1016/j.ydbio.2023.12.010 38159623 PMC10922954

[16] Halley J.E., Kaplan T., Wang A.Y., Kobor M.S., Rine J., 2010. Roles for H2A.Z and Its Acetylation in GAL1 Transcription and Gene Induction, but Not GAL1-Transcriptional Memory. PLOS Biol. 8, e1000401. 10.1371/journal.pbio.1000401 20582323 PMC2889906

[17] Hickey G.J., Wike C.L., Nie X., Guo Y., Tan M., Murphy P.J., Cairns B.R., 2022. Establishment of developmental gene silencing by ordered polycomb complex recruitment in early zebrafish embryos. eLife 11, e67738. 10.7554/eLife.67738 34982026 PMC8769650

[18] Hu G., Cui K., Northrup D., Liu C., Wang C., Tang Q., Ge K., Levens D., Crane-Robinson C., Zhao K., 2013. H2A.Z Facilitates Access of Active and Repressive Complexes to Chromatin in Embryonic Stem Cell Self-Renewal and Differentiation. Cell Stem Cell 12, 180–192. 10.1016/j.stem.2012.11.003 23260488 PMC3570599

[19] Ishibashi T., Dryhurst D., Rose K.L., Shabanowitz J., Hunt D.F., Ausió J., 2009. Acetylation of Vertebrate H2A.Z and Its Effect on the Structure of the Nucleosome. Biochemistry 48, 5007–5017. 10.1021/bi900196c 19385636 PMC2850812

[20] Jin C., Felsenfeld G., 2007. Nucleosome stability mediated by histone variants H3.3 and H2A.Z. Genes Dev. 21, 1519–1529. 10.1101/gad.1547707 17575053 PMC1891429

[21] Jin C., Zang C., Wei G., Cui K., Peng W., Zhao K., Felsenfeld G., 2009. H3.3/H2A.Z double variant–containing nucleosomes mark “nucleosome-free regions” of active promoters and other regulatory regions. Nat. Genet. 41, 941–945. 10.1038/ng.409 19633671 PMC3125718

[22] Kaya-Okur H.S., Wu S.J., Codomo C.A., Pledger E.S., Bryson T.D., Henikoff J.G., Ahmad K., Henikoff S., 2019. CUT&Tag for efficient epigenomic profiling of small samples and single cells. Nat. Commun. 10, 1930. 10.1038/s41467-019-09982-5 31036827 PMC6488672

[23] Kobor M.S., Venkatasubrahmanyam S., Meneghini M.D., Gin J.W., Jennings J.L., Link A.J., Madhani H.D., Rine J., 2004. A Protein Complex Containing the Conserved Swi2/Snf2-Related ATPase Swr1p Deposits Histone Variant H2A.Z into Euchromatin. PLOS Biol. 2, e131. 10.1371/journal.pbio.0020131 15045029 PMC374244

[24] Kouzarides T., 2007. Chromatin Modifications and Their Function. Cell 128, 693–705. 10.1016/j.cell.2007.02.005 17320507

[25] Krogan N.J., Keogh M.-C., Datta N., Sawa C., Ryan O.W., Ding H., Haw R.A., Pootoolal J., Tong A., Canadien V., Richards D.P., Wu X., Emili A., Hughes T.R., Buratowski S., Greenblatt J.F., 2003. A Snf2 Family ATPase Complex Required for Recruitment of the Histone H2A Variant Htz1. Mol. Cell 12, 1565–1576. 10.1016/S1097-2765(03)00497-0 14690608

[26] Ku M., Jaffe J.D., Koche R.P., Rheinbay E., Endoh M., Koseki H., Carr S.A., Bernstein B.E., 2012. H2A.Z landscapes and dual modifications in pluripotent and multipotent stem cells underlie complex genome regulatory functions. Genome Biol. 13, R85. 10.1186/gb-2012-13-10-r85 23034477 PMC3491413

[27] Lademann C.A., Renkawitz J., Pfander B., Jentsch S., 2017. The INO80 Complex Removes H2A.Z to Promote Presynaptic Filament Formation during Homologous Recombination. Cell Rep. 19, 1294–1303. 10.1016/j.celrep.2017.04.051 28514650

[28] Latrick C.M., Marek M., Ouararhni K., Papin C., Stoll I., Ignatyeva M., Obri A., Ennifar E., Dimitrov S., Romier C., Hamiche A., 2016. Molecular basis and specificity of H2A.Z–H2B recognition and deposition by the histone chaperone YL1. Nat. Struct. Mol. Biol. 23, 309–316. 10.1038/nsmb.3189 26974126

[29] Link S., Spitzer R.M.M., Sana M., Torrado M., Völker-Albert M.C., Keilhauer E.C., Burgold T., Pünzeler S., Low J.K.K., Lindström I., Nist A., Regnard C., Stiewe T., Hendrich B., Imhof A., Mann M., Mackay J.P., Bartkuhn M., Hake S.B., 2018. PWWP2A binds distinct chromatin moieties and interacts with an MTA1-specific core NuRD complex. Nat. Commun. 9, 4300. 10.1038/s41467-018-06665-5 30327463 PMC6191444

[30] Malone H.A., Roberts C.W.M., 2024. Chromatin remodellers as therapeutic targets. Nat. Rev. Drug Discov. 23, 661–681. 10.1038/s41573-024-00978-5 39014081 PMC11534152

[31] Mao Z., Pan L., Wang W., Sun J., Shan S., Dong Q., Liang X., Dai L., Ding X., Chen S., Zhang Z., Zhu B., Zhou Z., 2014. Anp32e, a higher eukaryotic histone chaperone directs preferential recognition for H2A.Z. Cell Res. 24, 389–399. 10.1038/cr.2014.30 24613878 PMC3975505

[32] Meng F.W., Murphy K.E., Makowski C.E., Delatte B., Murphy P.J., 2023. Competition for H2A.Z underlies the developmental impacts of repetitive element de-repression. Development 150, dev202338. 10.1242/dev.202338 37938830 PMC10651094

[33] Millar C.B., Xu F., Zhang K., Grunstein M., 2006. Acetylation of H2AZ Lys 14 is associated with genome-wide gene activity in yeast. Genes Dev. 20, 711–722. 10.1101/gad.1395506 16543223 PMC1413291

[34] Murphy K.E., Meng F.W., Makowski C.E., Murphy P.J., 2020. Genome-wide chromatin accessibility is restricted by ANP32E. Nat. Commun. 11, 5063. 10.1038/s41467-020-18821-x 33033242 PMC7546623

[35] Murphy P.J., Wu S.F., James C.R., Wike C.L., Cairns B.R., 2018. Placeholder Nucleosomes Underlie Germline-to-Embryo DNA Methylation Reprogramming. Cell 172, 993–1006.e13. 10.1016/j.cell.2018.01.022 29456083

[36] Mylonas C., Lee C., Auld A.L., Cisse I.I., Boyer L.A., 2021. A dual role for H2A.Z.1 in modulating the dynamics of RNA polymerase II initiation and elongation. Nat. Struct. Mol. Biol. 28, 435–442. 10.1038/s41594-021-00589-3 33972784

[37] Nair S.S., Kumar R., 2012. Chromatin remodeling in Cancer: A Gateway to regulate gene Transcription. Mol. Oncol., Cancer epigenetics 6, 611–619. 10.1016/j.molonc.2012.09.005 PMC3538127 23127546

[38] Obri A., Ouararhni K., Papin C., Diebold M.-L., Padmanabhan K., Marek M., Stoll I., Roy L., Reilly P.T., Mak T.W., Dimitrov S., Romier C., Hamiche A., 2014. ANP32E is a histone chaperone that removes H2A.Z from chromatin. Nature 505, 648–653. 10.1038/nature12922 24463511

[39] Papamichos-Chronakis M., Watanabe S., Rando O.J., Peterson C.L., 2011. Global Regulation of H2A.Z Localization by the INO80 Chromatin-Remodeling Enzyme Is Essential for Genome Integrity. Cell 144, 200–213. 10.1016/j.cell.2010.12.021 21241891 PMC3035940

[40] Pünzeler S., Link S., Wagner G., Keilhauer E.C., Kronbeck N., Spitzer R.M., Leidescher S., Markaki Y., Mentele E., Regnard C., Schneider K., Takahashi D., Kusakabe M., Vardabasso C., Zink L.M., Straub T., Bernstein E., Harata M., Leonhardt H., Mann M., Rupp R.A., Hake S.B., 2017. Multivalent binding of PWWP2A to H2A.Z regulates mitosis and neural crest differentiation. EMBO J. 36, 2263–2279. 10.15252/embj.201695757 28645917 PMC5538766

[41] Rada-Iglesias A., Bajpai R., Swigut T., Brugmann S.A., Flynn R.A., Wysocka J., 2011. A unique chromatin signature uncovers early developmental enhancers in humans. Nature 470, 279–283. 10.1038/nature09692 21160473 PMC4445674

[42] Raisner R.M., Hartley P.D., Meneghini M.D., Bao M.Z., Liu C.L., Schreiber S.L., Rando O.J., Madhani H.D., 2005. Histone Variant H2A.Z Marks the 5′ Ends of Both Active and Inactive Genes in Euchromatin. Cell 123, 233–248. 10.1016/j.cell.2005.10.002 16239142 PMC2039754

[43] Reilly P.T., Yu Y., Hamiche A., Wang L., 2014. Cracking the ANP32 whips: Important functions, unequal requirement, and hints at disease implications. Bioessays 36, 1062–1071. 10.1002/bies.201400058 25156960 PMC4270211

[44] Ren Q., Gorovsky M.A., 2001. Histone H2A.Z Acetylation Modulates an Essential Charge Patch. Mol. Cell 7, 1329–1335. 10.1016/S1097-2765(01)00269-6 11430834

[45] Ruhl D.D., Jin J., Cai Y., Swanson S., Florens L., Washburn M.P., Conaway R.C., Conaway J.W., Chrivia J.C., 2006. Purification of a Human SRCAP Complex That Remodels Chromatin by Incorporating the Histone Variant H2A.Z into Nucleosomes. Biochemistry 45, 5671–5677. 10.1021/bi060043d 16634648

[46] Ryan D.P., Tremethick D.J., 2018. The interplay between H2A.Z and H3K9 methylation in regulating HP1α binding to linker histone-containing chromatin. Nucleic Acids Res. 46, 9353–9366. 10.1093/nar/gky632 30007360 PMC6182156

[47] Shi L., Wen H., Shi X., 2017. The Histone Variant H3.3 in Transcriptional Regulation and Human Disease. J. Mol. Biol., Deciphering Histone Modifications in Development and Disease 429, 1934–1945. 10.1016/j.jmb.2016.11.019 PMC5446305 27894815

[48] Skvortsova K., Iovino N., Bogdanović O., 2018. Functions and mechanisms of epigenetic inheritance in animals. Nat. Rev. Mol. Cell Biol. 19, 774–790. 10.1038/s41580-018-0074-2 30425324

[49] Subramanian V., Mazumder A., Surface L.E., Butty V.L., Fields P.A., Alwan A., Torrey L., Thai K.K., Levine S.S., Bathe M., Boyer L.A., 2013. H2A.Z Acidic Patch Couples Chromatin Dynamics to Regulation of Gene Expression Programs during ESC Differentiation. PLOS Genet. 9, e1003725. 10.1371/journal.pgen.1003725 23990805 PMC3749939

[50] Suto R.K., Clarkson M.J., Tremethick D.J., Luger K., 2000. Crystal structure of a nucleosome core particle containing the variant histone H2A.Z. Nat. Struct. Biol. 7, 1121–1124. 10.1038/81971 11101893

[51] Thambirajah A.A., Dryhurst D., Ishibashi T., Li A., Maffey A.H., Ausió J., 2006. H2A.Z Stabilizes Chromatin in a Way That Is Dependent on Core Histone Acetylation*. J. Biol. Chem. 281, 20036–20044. 10.1074/jbc.M601975200 16707487

[52] Tochio N., Umehara T., Munemasa Y., Suzuki T., Sato S., Tsuda K., Koshiba S., Kigawa T., Nagai R., Yokoyama S., 2010. Solution Structure of Histone Chaperone ANP32B: Interaction with Core Histones H3–H4 through Its Acidic Concave Domain. J. Mol. Biol. 401, 97–114. 10.1016/j.jmb.2010.06.005 20538007

[53] Weber C.M., Ramachandran S., Henikoff S., 2014. Nucleosomes Are Context-Specific, H2A.Z-Modulated Barriers to RNA Polymerase. Mol. Cell 53, 819–830. 10.1016/j.molcel.2014.02.014 24606920

[54] Willhoft O., Wigley D.B., 2020. INO80 and SWR1 complexes: the non-identical twins of chromatin remodelling. Curr. Opin. Struct. Biol., Theory and Simulation ● Macromolecular Assemblies 61, 50–58. 10.1016/j.sbi.2019.09.002 PMC7171469 31838293

[55] Wu R.S., Tsai S., Bonner W.M., 1982. Patterns of histone variant synthesis can distinguish go from G1 cells. Cell 31, 367–374. 10.1016/0092-8674(82)90130-1 7159927

[56] Yen K., Vinayachandran V., Pugh B.F., 2013. SWR-C and INO80 Chromatin Remodelers Recognize Nucleosome-free Regions Near +1 Nucleosomes. Cell 154, 1246–1256. 10.1016/j.cell.2013.08.043 24034248 PMC4090706

[57] Yu J., Sui F., Gu F., Li W., Yu Z., Wang Q., He S., Wang L., Xu Y., 2024. Structural insights into histone exchange by human SRCAP complex. Cell Discov. 10, 15. 10.1038/s41421-023-00640-1 38331872 PMC10853557

[58] Zlatanova J., Thakar A., 2008. H2A.Z: View from the Top. Structure 16, 166–179. 10.1016/j.str.2007.12.008 18275809

